# Efficacy of Caltropis procera and Ficus sycomorus extracts in treating MRSA (methicillin-resistant Staphylococcus aureus)-keratitis in rabbit

**DOI:** 10.17179/excli2015-350

**Published:** 2015-06-16

**Authors:** Waiel F. Sayed, Wesam M. A. Salem, Mohie A. M. Haridy, Ne'mat H. Hassan

**Affiliations:** 1 Department of Botany, Faculty of Science, South Valley University, 83523 Qena, Egypt; 2 Department of Pathology and Clinical Pathology, Faculty of Veterinary Medicine, South Valley University, 83523 Qena, Egypt; 3 The Egyptian Organization for Standardization and Quality, Cairo, Egypt

**Keywords:** Ficus sycomorus, Caltropis procera, latex, Staphylococcus aureus MRSA, keratitis

## Abstract

MRSA-induced keratitis in rabbit was used to evaluate the therapeutic effect of *F. sycomorus* leaves and *C. procera* latex extracts. Within the 6 rabbit groups tested, group 1 received sterilized saline, while other groups (2 to 6) received 100 μl of intrastromal injections of 1.5×10^3^ colony forming unit (cfu) ml^-1^ of methicillin-resistant *Staphylococcus aureus* (MRSA). After 12 hours, groups 3 to 6 also received chloramphenicol, aqueous extract of *C. procera* latex, aqueous and alcoholic extracts of *F. sycomorus* leaves, respectively 3 times daily for 12 successive days. The tested extracts inhibited MRSA growth *in vitro* (i.e. on culture medium). Colony counts in cornea discs from groups 3 to 6 were significantly reduced (*P *≤ 0.001) compared to group 2 (untreated). Clinical signs of keratitis were observed on group 2 until the end of experiment. In groups 3 to 6, gradual recovery was observed and signs disappeared by the 12^th^ DPI (days post inoculation). Only mild symptoms persisted in group 5 (aqueous extract of leaves). In group 3 and 5, cornea, iris, ciliary body and conjunctiva showed mild leukocytic infiltration and depigmentation of melanin cells while recovery of cornea and iris was observed in groups 4 and 6. In conclusion, the used extracts have potential therapeutic effects on MRSA-induced keratitis in rabbit.

## Introduction

Bacterial keratitis is one of the most visually threatening ocular infections due to its potential complications (Bourcier et al., 2003[4]). Bacterial infection of the cornea occurs in a two-step process: 1) bacteria interact with the surface of corneal epithelial cells and then, 2) penetrate into the stroma where toxins mediate severe inflammation and tissue damage (O'Callaghan et al., 1999[22]). Corneal perforations occur in less than 24 h in the presence of both *Pseudomonas aeruginosa* and *Staphylococcus* *aureus* (Schaefer et al., 2001[31]). If appropriate therapy is not promptly initiated, these bacteria can proliferate rapidly through the production of enzymes such as proteases, coagulases, collagenases and lipases with exotoxins that facilitate corneal tissue necrosis and aid in deep penetration into the stroma. This will cause a rapid destructive infection that can lead to loss of the entire eye (O'Brien, 2003[20]).

*Staphylococcus* *aureus* is a major cause of bacterial keratitis and is responsible for various community onset and hospital acquired infections. *Staphylococcus aureus *ocular infections can cause severe inflammation, pain, corneal perforation, scarring, and loss of visual sharpness (Gordon and Lowy, 2008[9]; Ippolito et al., 2010[11]). Genetic, immunologic, and histopathologic studies have shown that the major cause of these pathological events is the action of α-toxin that is a lytic toxin produced by *Staphylococcus* *aureus* in the late log phase of growth (O'Callaghan et al., 1997[21]). Antibiotic resistant *Staphylococcus* strains are of major public health concern since the bacteria can easily circulate in the environment and because *Staphylococcus aureus *has a long history of evolving continuously to more resistant states (Yamamoto et al., 2010[34]). Therefore, new antibiotic formulations are needed to manage future cases of *Staphylococcus aureus* induced keratitis. At present, the drugs of choice against *Staphylococcus aureus* infections are the β-lactam antibiotics. Unfortunately,* Staphylococcus aureus *has developed resistance to the β-lactam antibiotics due to synthesis of chromosomal or plasmid-encoded β-lactamases (Wertheim et al., 2005[33]). In comparison to methicillin-sensitive *Staphylococcus aureus *(MSSA), methicillin-resistant *Staphylococcus aureus *(MRSA) strains pose more problems, since invasive MRSA infections are associated with greater costs and limited treatment options (Cosgrove et al., 2005[6]).

Induction of keratitis is either by contact lens injury or by intrastromal infection. Host defenses were so effective for a scarified cornea topically inoculated with 10 million colony forming units (cfu's) of *Staphylococcus aureus* adhering to a contact lens. This inoculation failed to cause a productive infection and resulted in efficient bacterial killing within the tear film (Hume et al., 2001[10]). This killing effect is in contrast to the extensive bacterial replication and pathologic effect of keratitis after intrastromal injection of bacteria into the cornea (Hume et al., 2001[10]). Hence, the intrastromal model of *Staphylococcus* keratitis is more useful for studying the chemotherapy and pathological reactions that occur in the eye. New antibacterial substances of different origins are being experimented and the use of natural antibacterial substances from bacteria, plants and animals are recently hot research topics (Kurlenda and Grinholc, 2012[14]; Salem et al., 2014[28][29], 2015[27]).

In the current study, aqueous and 70 % ethanol extracts of *F. sycomours *leaves and aqueous extract of *C. procera* latex were investigated for their *in vitro* and *in vivo* efficacy in treating experimentally intrastromal MRSA-induced keratitis in rabbit. 

## Materials and Methods

### Plant materials and preparation of the extracts

*F. sycomorus* leaves and *C. procera *latex were collected from Botany farm, Faculty of Science, South Valley University, Qena, Egypt. White milky latex was collected in sterile bottles after wounding the green stem. To obtain aqueous extracts, ten grams of air-dried powdered *F. sycomorus* leaves and *C. procera* latex, were dissolved in 100 ml sterile distilled water under shaking conditions (180 rpm) for 5 days. The extracts were filtered through Whatman No. 1 filter paper and used as a medium for preparing sodium phosphate buffer (Verástegui et al., 1996[32]). For alcoholic extract of dried leaves, 10 gm were dissolved in 100 ml of 70 % ethanol. The filtrate was evapourated to dryness at room temperature and the residue was dissolved in sodium phosphate buffer to prepare the required concentration (Verástegui et al., 1996[32]).

### Kinetic study of the extracts

The clinical isolate methicillin- and oxacillin-resistant *Staphylococcus aureus* (MRSA, ATCC43300) was incubated overnight at 37 °C in tryptic soy broth (TSB) (Mac Faddin, 1985[15]). After shaking, 5 ml of overnight broth culture of MRSA was mixed with 25 ml fresh Mueller-Hinton broth (Muller and Hinton, 1941[18]) and 1 ml of the tested extracts. Control tubes received 1 ml sterile distilled water instead of plant extracts. The second control (blank) contained 29 ml fresh Mueller-Hinton broth and 1 ml of each corresponding extract. Optical density of the different treatments was measured at 427 nm every 30 minutes for five hours (Ali et al., 2001[1]) using a “Spectronic® Genesys™ 2PC” Spectrophotometer, Spectronic Instruments, USA.

### In vivo studies

Growth of MRSA, grown overnight at 37 °C in TSB medium, was diluted in the same medium to give approximately 1.5 x 10^3^ cfu ml^-1^.

Eighteen local bread (Baladi) rabbits (2 months old), with average weight between 1 and 1.5 kg, were used for intrastromal injection to induce MRSA keratitis according to ”South Valley University scientific ethics and animal care and use guidelines”. Animals were housed in standard cages and kept in a controlled room conditions at 25 ± 1 °C and 50 ± 5 % relative humidity, with continuous medical care and observation until the end of experimentation.

### Rabbit corneal inoculation

Animals were divided into six groups; group 1 received normal saline solution as control and groups 2 to 6 received only one time intrastromal injection of MRSA. Local anesthetic treatment of aureculopalbebral nerve was applied with 0.1 ml lidocaine HCL 2 % (Sigmatec Pharmaceutical Industries, Giza, Egypt). Each cornea was injected with a 100 μl saline solution containing approximately 1500 cfu of MRSA using a 30-gauge needle (Callegan et al., 1992[5]). Twelve hours post injection, all animals exhibited clinical signs of bacterial keratitis. Group 2 (infected but untreated rabbits) received no treatment over the experimental period. Groups 3, 4, 5 and 6 received treatments every 8 h for 12 days using two drops (approx. 10 µl) of chloramphenicol (30 mg), aqueous extract of *C. procera *latex and aqueous and ethanol extracts of *F. sycomorus *leaves, respectively. Treatments for different groups are summarized in Table 1(Tab. 1). The groups were examined by naked eye on the 5^th^, 9^th^, and 12th day post-inoculation (DPI) for clinical signs of bacterial keratitis (blepharitis, iritis, conjunctivitis and corneal oedema). The observed clinical signs were scored collectively by two veterinary ophthalmologists on severity scale from 0 to 3 and total score for each eye was determined (Romanowski et al., 2005[26]).

After aseptical dissection of corneas, 1 cm button was removed and homogenized in sterile saline phosphate buffer (0.9 % NaCl) by a tissue homogenizer. Aliquots of corneal homogenates were serially diluted in buffered saline, plated in triplicate on Mueller-Hinton agar containing 5 % sheep blood, and incubated at 37 °C for 48 h. The number of MRSA per cornea was counted using a colony counter (Romanowski et al., 2005[26]; Dajcs et al., 2004[7]).

### Histopathological study

The other portions of corneas were prepared for microscopical examination according to Moreau et al. (1997[16]) by fixing immediately in 10 % neutral buffered formalin, followed by dehydration in upgraded concentrations of alcohol and immersion in xylene until clearance. Corneal tissues were then embedded in paraffin wax and the prepared blocks were cut into 4 mm thick sections using Leica™ RM2235 rotary microtome (Germany). Sections were stained with hematoxylin and eosin and observed under brightfield microscopy for tissue abnormalities.

### Statistical analysis

Each data set represents the mean and standard deviation (± SD) from at least three independent experiments. The clinical signs were analyzed using Kruskal Wallis ANOVA with Duncan's multiple comparisons. The colony counts from three animals were log-transformed and analyzed parametrically using ANOVA. Significant differences between the data sets are marked by different letters (*P ***<** 0.05). The other statistical analyses were performed by using "GraphPad Prism version 5". 

## Results

### In vitro effect of plant extracts on MRSA culture

**Figure 1:**
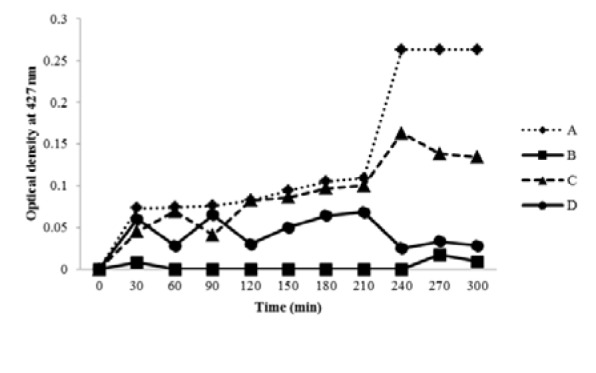
Antibacterial activities of *C. procera* and *F. sycomoru*s extracts against *Staphylococcus aureus *MRSA. A: Control (untreated culture); B: aqueous extract of *F. sycomorus*; C: alcoholic extract of *F. sycomorus*; D: aqueous extract of *C. procera* latex.

The inhibitory effects of different plant extracts on MRSA growth were variable (Figure 1(Fig. 1)). Aqueous extracts of *C. procera* latex and *F. sycomorus* leaves were more inhibitory than alcoholic extract of *F. sycomorus* leaves. The optical density of control bacterial culture at 427 nm gradually increased until reaching the maximum growth level after 240 minutes, then fixed until reaching 300 minutes. However, optical densities of cultures containing the plant extracts gradually decreased. Alcoholic extract of *F. sycomorus* leaves is suggested to have a bacteriostatic effect while aqueous extracts of *C. procera* latex and *F. sycomorus* leaves exhibited bactericidal effects (Figure 1(Fig. 1)).

### Clinical signs

**Figure 2:**
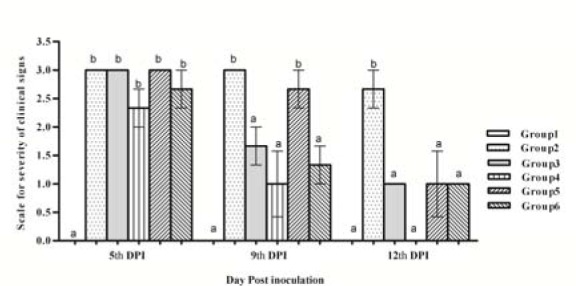
Severity of clinical signs of *Staphylococcus aureus*-MRSA keratitis in rabbit model treated with extracts of *F. sycomarus* and *C. procera*. Values marked with the same letters (ex; a-a) were not significantly different; values marked with different letters (ex; a-b) were considered statistically significant (P< 0.05) according to Duncan's Test.

Symptoms of bacterial keratitis were graded as previously described and total score for each animal's eye was collectively determined. Animals of group (1) did not show any clinical signs throughout the experiment. At the 5^th^ DPI, severe clinical signs were observed in groups 2 and 5 and moderate signs in groups 3, 4 and 6. At the 9^th^ DPI, severe signs were still existed in groups 2 (untreated) but were less severe in group 5 (aqueous extract of *F. Sycomorus* leaves). Mild clinical signs were observed in the eyes of group 3 and 6, but no signs of eye inflammation were observed in group 4 (latex). At the 12^th^ DPI, severe signs of keratitis were still observed in group 2 while mild signs were observed in groups 3 and 5. No clinical signs were observed at all in groups 4 (aqueous extract of *C. Procera* latex) and 6 (ethanol extract of *F. Sycomorus* leaves). The scores for bacterial keratitis clinical signs and the treated groups are shown in Figure 2(Fig. 2). The scores of clinical signs were not siginficantly different in groups 3 to 6 from group 2 at the 5^th^ DPI. In contrast, they decreased significantly in group 3, 4 and 6 at the 9^th^ DPI. At the 12^th^ DPI, in all treated groups (3 to 6), the scores of clinical signs were reduced significantly (recovery progress) to a level almost similar to the control group (Figure 2(Fig. 2)).

### Microbiological load of the corneal tissue discs

**Figure 3:**
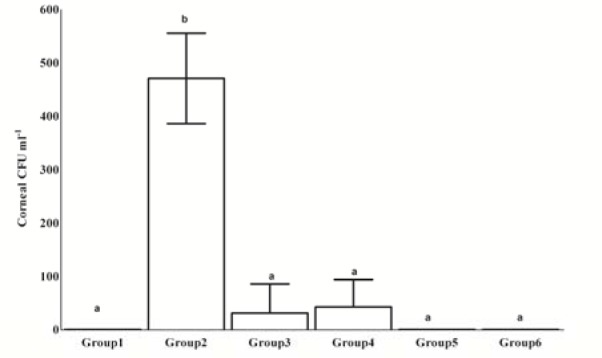
Total colony counts from corneal discs of different groups after 12 days of treatment. Values marked with the same letters (ex; a-a) were not significantly different; values marked with different letters (ex; a-b) were considered statistically significant (P ≤ 0.001) according to Duncan's Test.

Results of the microbiological loads of corneas are presented in Figure 3(Fig. 3). Total colony counts (cfu ml^-1^) for each group were determined by calculating the average of three treated corneas. The colony count was 4.7 ± 8.5 log_10_ cfu ml^-1^ in group 2 (untreated) and 0.3 ± 0.5 log_10_ cfu ml^-1^ in groups 3 (chloramphenicol-treated). Corneas of group 4 recorded 0.1 ± 0.2 log_10_ cfu ml^-1^ (aqueous extract of *C. Procera* latex). Finally, corneas of groups 5 and 6 yeilded no bacterial counts (aqueous and ethanol extracts of *F. Sycomorus* leaves).

Treatment of rabbit eyes in groups 3, 4, 5 and 6 significantly reduced total colony counts (*P *≤ 0.001) in comparison to the untreated MRSA-inoculated group (group 2). The *Staphylococcus aureus *load was approximately reduced 4 to 5 log of cfu/cornea when compared to group 2 (Figure 3(Fig. 3)). No significant differences were observed in the numbers of cfu per cornea among the treated groups from 3 to 6.

### Histopathological study

**Figure 4:**
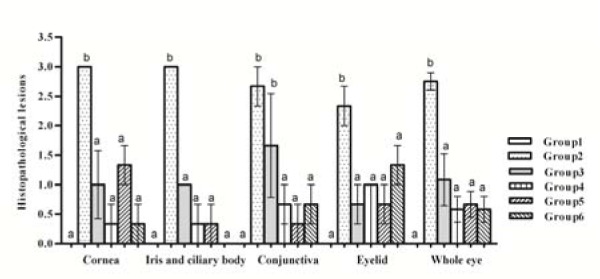
Scores of the histopathological lesions of MRSA-induced keratitis in rabbit groups after 12 days of different treatments. Values marked with the same letters (ex; a-a) were not significantly different; values marked with different letters (ex; a-b) were considered statistically significant (P < 0.05) according to Duncan's Test.

The histopathological lesions in different treatments are summarized in Table 2(Tab. 2) and Figure 4(Fig. 4).

In general, no abnormalities were observed in group (1) for cornea, ciliary body, iris, conjunctiva or eyelids. For the other groups, the observed signs in the prepared sections were as follows:

**Cornea: **In group 2, severe suppurative keratitis, conjunctivitis and iriditis were observed. The recorded changes were necrosis and ulceration of the corneal epithelium with intensive neutrophils cell infiltration. The underlying loose connective tissue was heavily infiltrated with neutrophils. The stroma appeared dispersed and also heavily infiltrated with neutrophils with focal areas of necrosis in the stroma. Heavy leukocytic cell infiltration and newly formed blood vessels were observed near the Descemet's membrane with parts of iris adhered to it. The corneal stromas were mild edematous and inflammatory cells were sparsely infiltrated in group 3. For the same group, the junction between bulbar conjunctiva, cornea and limbus revealed that the lamina propria, underlying the conjunctiva, is edematous and sparsely infiltrated with leukocytes. Infiltration of sclera with leukocytes and the base of cilliary body was observed. The corneas were intact without any inflammatory cell infiltration in groups 4 and 5, with the corneas almost intact in 2 out of 3 cases. The third case showed mild leukocytic cell infiltration in the sub-epithelium. In group 6, the corneas were intact with no inflammatory lesions and with minimal leukocytic cells infiltration near the Descemet's membrane.

**Iris and ciliary body: **In group 2, the iris was thickened and heavily infiltrated with eosinophilic proteinaceous material, neutrophils and macrophages with desquamation and depletion of the iridal lining pigmented epithelium and profound diffuse depigmentation. Cytoplasmic vacuolation, shrinking of the nucleus and absence of melanin cells of the iris stroma were observed. In group 3, desquamation and depletion of the iridal and ciliary processes lining the pigmented epithelium (with profound diffuse depigmentation) was observed. In group 4, the iris and ciliary body were lined by pigmented epithelium and melanocytes in the stroma. The iridal and ciliary processes lining with pigmented epithelium were still intact but with decreased melanocytes in the iris stroma in group 5. In group 6, the iris and ciliary body were intact with almost normal pigmentation.

**Conjunctiva and eyelids: **In group 2, diffuse leukocytic cell infiltration in the palpebral conjunctiva with intra-epithelial migratory neutrophil resulted in destruction and necrosis of the lining epithelium with submucosal leukocytic infiltration. The eyelid had severe neutrophil cell infiltration in the cutaneous epithelium and adnexa. In group 3, thickening of the conjunctival mucosa with intensive leukocytic infiltration was observed in the lining epithelium and the lamina propria. In group 4, the loose connective tissue beneath the conjunctival mucosa and eyelids were minimally infiltrated with leukocytes. In group 5, the lamina propria of the conjunctiva, just before transition to corneal epithelium, was infiltrated with leukocytes. The conjunctiva and eyelids were heavily infiltrated with leukocytes in 1 out of 3 cases with the other two cases mildly infiltrated with leukocytes. In group 6, minimal mononuclear cell infiltration in conjunctiva and eyelid was observed.

The scores of the histopathological lesions of cornea, iris, ciliary body, conjunctiva and eyelid, in different animals of groups 3 to 6, were significantly lower than those of group 2. Generally, the score was not significantly different among the treated groups from 3 to 6. The mean of the total scores for each eye was summed as the whole eye score that was significantly reduced in groups 3 to 6 compared to group 2 (Figure 4(Fig. 4)).

For more details of the histopathological and microscopical observations, see the supplementary Figures 1-4.

## Discussion

The eye is a unique organ that is virtually impermeable to most environmental agents. Continuous tear flow, aided by the reflex blinking, provides lubrication to wash away substances from the ocular surface and prevents the accumulation of microorganisms (Kaufman et al., 1988[12]). Tears also contain some antimicrobial agents including lymphocytes, immunoglobulins, lysozyme and lactoferrin that specifically reduce bacterial colonization of the ocular surface (Kaufman et al., 1988[12]). However, predisposing factors such as corneal injury, allergic hypersensitivity reactions, corneal abnormalities and overuse of contact lenses may alter the defense mechanisms of the ocular surface and permit bacteria to invade the cornea causing epithelial defects (Bourcier et al., 2003[4]).

In the current study, the inhibitory effect of *F. sycomorus* aqueous extract on MRSA growth was more pronounced than its alcoholic extract. The effect of *F. sycomorus* alcoholic extract was bacteriostatic while it was bactericidal for the aqueous extracts of *F. sycomorus* leaves and *C. procera* latex. The leaves of *F. sycomorus* were reported to contain calcium, phosphorous, iron, magnesium and zinc. The stem bark extract contains tannins, saponins, reducing sugars, flavones, aglycones, anthraquinone glycosides, flavonoid glycosides and condensed tannins that have an inhibitory effect on both smooth and skeletal muscle contractions (Sandabe et al., 2006[30]).

Extracts of *C. procera* using water, methanol, absolute ethanol, and ethanol 70 % were effective against MRSA (Salem et al., 2014[29]). The apical twigs and latex of *C. procera* were reported to produce the greatest inhibition zones against *Staphylococcus aureus* (Parabia et al., 2008[24]). The bactericidal activity of *C. procera* latex could be due to the presence of calactin, mudarin and the protein calotropain as active constituents (Moustafa et al., 2010[17]). 

In the current study, symptoms of bacterial keratitis such as blepharitis, conjunctivitis, iritis and corneal edema are the most important signs appeared after 12 h of MRSA inoculation. The scores of keratitis clinical signs did not significantly change in groups 3 to 6 when compared to group 2 at the 5^th^ DPI. In contrast, they significantly decreased in group 3, 4 and 6 at the 9^th^ DPI. Moreover, in all treated groups (3 to 6), the scores of clinical signs were significantly reduced and the eye recovery was comparable to the control group levels at the 12^th^ DPI (Figure 2(Fig. 2)). Treatments of rabbit eyes by *F. sycomorus *and *C. procera* extracts reduced the number of bacterial cfu/cornea approximately 4 to 5 log compared to the untreated control group (*P *≤ 0.001) as shown in Figure 3(Fig. 3). No significant differences were observed in the cfu/ cornea among groups 3 (chloramphenicol-treated) and 4, 5 and 6 (treated with extracts of *C. procera *and* F. sycomorus*). There is a correlation between the *in vitro* reduction of the optical densities in MRSA culture, treated with the extracts of *C. procera *and* F. sycomorus*, and the *in vivo* efficacy of these extracts on bacterial keratitis. The inhibitory effect, on MRSA* in vitro*, was exhibited *in vivo* by killing the bacterium in the early stage of infection by aqueous extracts of *C. procera* latex and *F. sycomorus* leaves and alcoholic extract of *F. sycomorus* leaves. On the other hand, flavonoids (a major constituent of all extracts) directly inhibit the bacterial growth and their virulence mechanisms (Oh et al., 2011[23]). *F. sycomorus* and *C. procera* are rich in flavonoids and phenolics as reported earlier (Nenaah, 2013[19]).

The rabbit model of bacterial keratitis induced through intrastromal injection is a reproducible manner that does not require special instruments and is preferred than contact lens injury method. Moreover, injection of only 100 cfu's of *Staphylococcus aureus* into the corneal stroma results in severe keratitis, because the stroma does not have the host defense systems found in the tear film (O'Callaghan et al., 1999[22]). Keratitis resulting from intrastromal injection is characterized by bacterial replication and severe ocular changes, including corneal edema, corneal epithelial cell destruction, and other symptoms (O'Callaghan et al., 1997[21], 1999[22]). The severe destructive keratitis in the current study, observed in group 2, is similar to those previous models of intrastromal injection of keratitis (O'Callaghan et al., 1999[22]). Clinical signs, total bacterial count and histopathological findings of bacterial keratitis in group 2 are due to severe tissue damage and inflammation produced by the action of specific toxins secreted by MRSA. These toxins are produced primarily after the bacteria have completed rapid growth cycles (O'Callaghan et al., 1997[21]). The implication of the rabbit model on clinical ophthalmology is that a patient with significant clinical signs of keratitis has a large population of toxin-producing, slowly replicating bacteria. In the present study, once considerable inflammation and tissue damage occurred in the rabbit, 12h post-injection of MRSA, effective killing of bacteria and inhibition of replication occurred by using extracts of *C. procera* latex and *F. sycomorus* leaves.

To assess the efficacy of *C. procera* latex and *F. sycomorus* leaves extracts they were compared to the effect of the antibiotic chloramephnicol that is broadly used in treating ocular infections. Animals of group 2 showed several symptoms and severe inflammation in the anterior chamber. The symptoms of MRSA keratitis were progressively getting worse with the increase in time unless properly treated (Ali et al., 2001[1]; Nenaah, 2013[19]), that was observed in group 2 over the period of study (12 days) as shown in Figures 2-4(Fig. 2)(Fig. 3)(Fig. 4). In contrast, with the used treatments, cornea, iris and ciliary body and conjunctiva had mild leukocytic infiltration and depigmentation of melanin cells in group 3 and 5. More effective treatments were revealed by complete recovery of corneal epithelium and iridial pigmentation in groups 4 and 6.

Keratitis was reported to develop some scars, neovascularization and some abscesses on day 14 in the eyes treated with 0.03 % vancomycin ointment indicating an active infection (Rasik et al., 1999[25]). Even if an infectious keratitis is treated adequately, it can cause a scar in the cornea within 2 weeks after disease manifestation (Eguchi et al., 2009[8]). In the current study, the conjunctiva and corneas appeared with intact epithelium and no prominent cellular infiltration in groups 4 and 6 that might be due to the healing effect, anti-inflammatory and antibacterial activity of *F. sycomorus* and *C. procera* extracts. One or more of the extract constituents is suggested to be responsible for that healing effect. Determination of the precise concentrations as well as the effective component(s) require further investigations. Latex of ***C. procera*** significantly augmented the healing process of dermal wounds in Guinea pigs by markedly increasing collagen, DNA and protein synthesis and epithelialization leading to reduction in wound area (Arya and Kumar, 2005[3]). Anti-inflammatory effect of *C. procera* latex aqueous extract was also approved (Kumar and Basu, 1994[13]). Moreover, procerain, a stable cysteine protease, is a component of the extract of *C. procera* latex that might diffusely infiltrate into cornea with bactericidal activity against *Staphylococcus aureus* leading to recovery (as in group 4) as reported earlier (Nenaah, 2013[19]). The fruit extract of Egyptian *Ficus* species, including *F. sycomorus*, were also reported for its significant antibacterial activity (Arunachalam and Parimelazhagan, 2013[2]).

In conclusion, the aqueous and alcoholic extracts of *F. sycomorus* and *C. procera* latex and leaves were potentially effective in the treatment of MRSA-induced keratitis in rabbit. Therefore, these extracts are suggested as efficient keratitis therapeutic agents in the correct doses that should be further investigated side by side with its natural components that exhibit and improve the healing effects of these extracts. 

## Acknowledgement

Dr. Madeh Sadan, Lecturer, Faculty of Veterinary Medicine, South Valley University is appreciated for his help in intrastromal injection and evaluation of the clinical signs. The participation of Dr. Soha Soliman, lecturer in the same Faculty, is thankfully appreciated for sectioning and staining of the histopathological samples.

## Conflict of interest statement

The authors declare that they have no conflict of interest.

## Ethical standards

This work was approved by South Valley University animal care and use committee (Approval No. 12-214). The use and care of experimental animals, used in this study, comply with the Egyptian animal welfare laws and policies.

## Supplementary Material

Supplementary material

## Figures and Tables

**Table 1:**
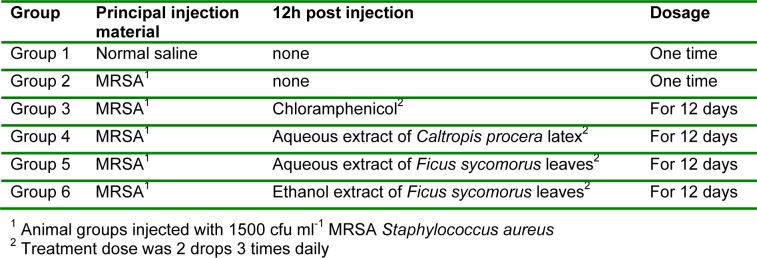
Injection material, treatment time and dosages for different groups

**Table 2:**
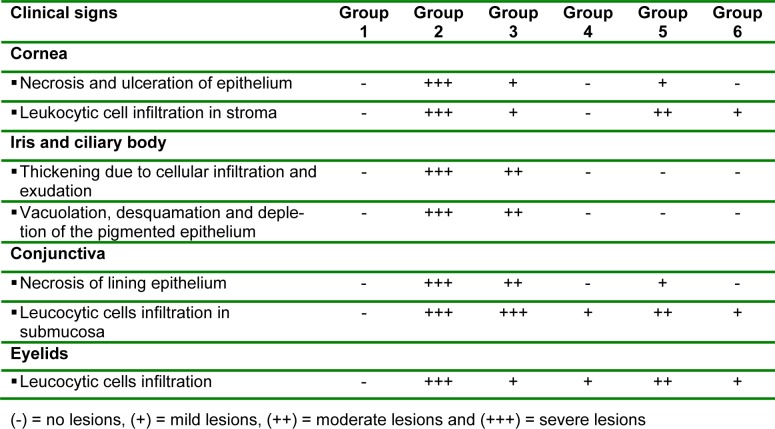
The histopathological lesions of MRSA-keratitis in rabbit model after 12 days of different treatments
