# Supplementary material for: Efficacy of Caltropis procera and Ficus sycomorus extracts in treating MRSA (methicillin-resistant Staphylococcus aureus)-keratitis in rabbit
Source: EXCLI J. 2015 Jun 16;14:747–57. doi: 10.17179/excli2015-350 (PMC4669905; doi:10.17179/excli2015-350)
Supplement: Supplementary material [file EXCLI-14-747-s-001.pdf]

EXCLI Journal 2015;14:747-757 – ISSN 1611-2156
Received: April 05, 2015, accepted: May 20, 2015, published: June 16, 2015

Suppplementary material to

EFFICACY OF *CALTROPIS PROCERA*AND *FICUS SYCOMORUS* EXTRACTS IN TREATING MRSA (METHICILLIN-RESISTANT *STAPHYLOCOCCUS AUREUS)*-KERATITIS IN RABBIT

Waiel F. Sayed[1*], Wesam M. A. Salem[1], Mohie A. M. Haridy[2], Ne'mat H. Hassan[3]

[1] Department of Botany, Faculty of Science, South Valley University, 83523 Qena, Egypt
[2] Department of Pathology and Clinical Pathology, Faculty of Veterinary Medicine, South Valley University, 83523 Qena, Egypt
[3] The Egyptian Organization for Standardization and Quality, Cairo, Egypt

* Corresponding author:Professor of Microbiology, Department of Botany, Faculty of Science, South Valley University, Qena, Egypt, E-mail: farghaly11@lycos.com

http://dx.doi.org/10.17179/excli2015-350

This is an Open Access article distributed under the terms of the Creative Commons Attribution License (http://creativecommons.org/licenses/by/4.0/).

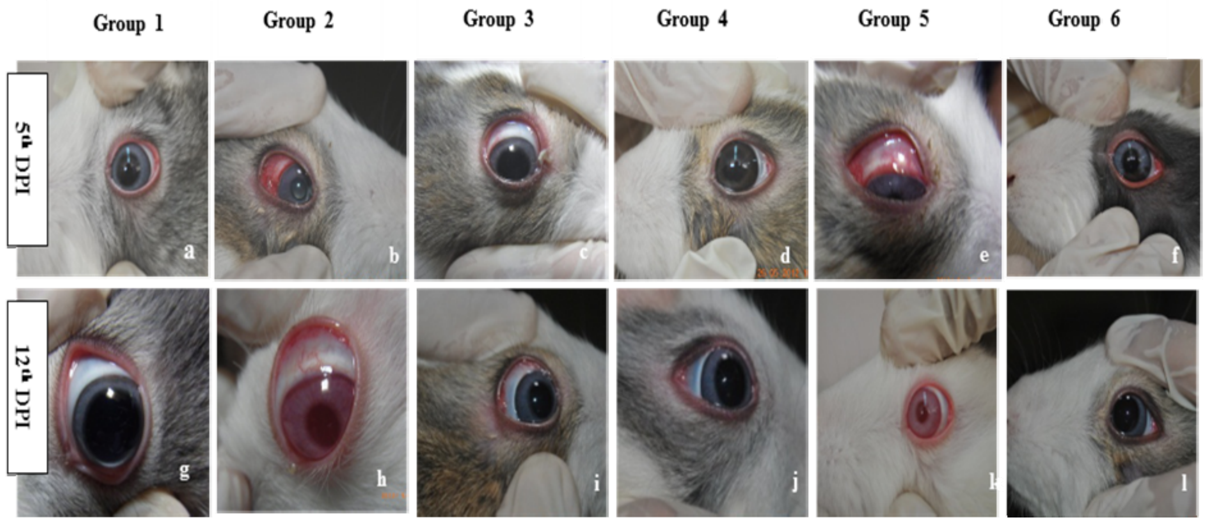

Supplementary Figure 1: Clinical signs of bacterial keratitis after treatment with extracts of *C. procera latex* and *F. sycomorus* leaves. Group 1 (a, g). received sterilized saline solution served as control (normal eyes view, with no clinical signs symptoms); groups from 2 to 6 were injected with 100 µl saline solution containing approximately 1500 cfu of MRSA (*S. aureus*). At the 5th DPI, severe clinical signs were observed in groups 2 (b) (untreated) and 5 (e) (treated with aqueous extract of *F. sycomorus* leaves) and moderate signs in groups 3 (c) (treated with chloramephnicol), 4 (d) (treated with aqueous extract of *C. procera* latex) and 6 (f) (treated with ethanol extract of *F. sycomorus* leaves). At the 12th DPI, severe signs of keratitis were still observed in group 2 (h) while mild signs were observed in groups 3 (i) and 5 (k). No clinical signs were observed in groups 4 (j) and 6 (l). In all treatments, the dose was 2 drops 3 times daily.

EXCLI Journal 2015;14:747-757 – ISSN 1611-2156
Received: April 05, 2015, accepted: May 20, 2015, published: June 16, 2015

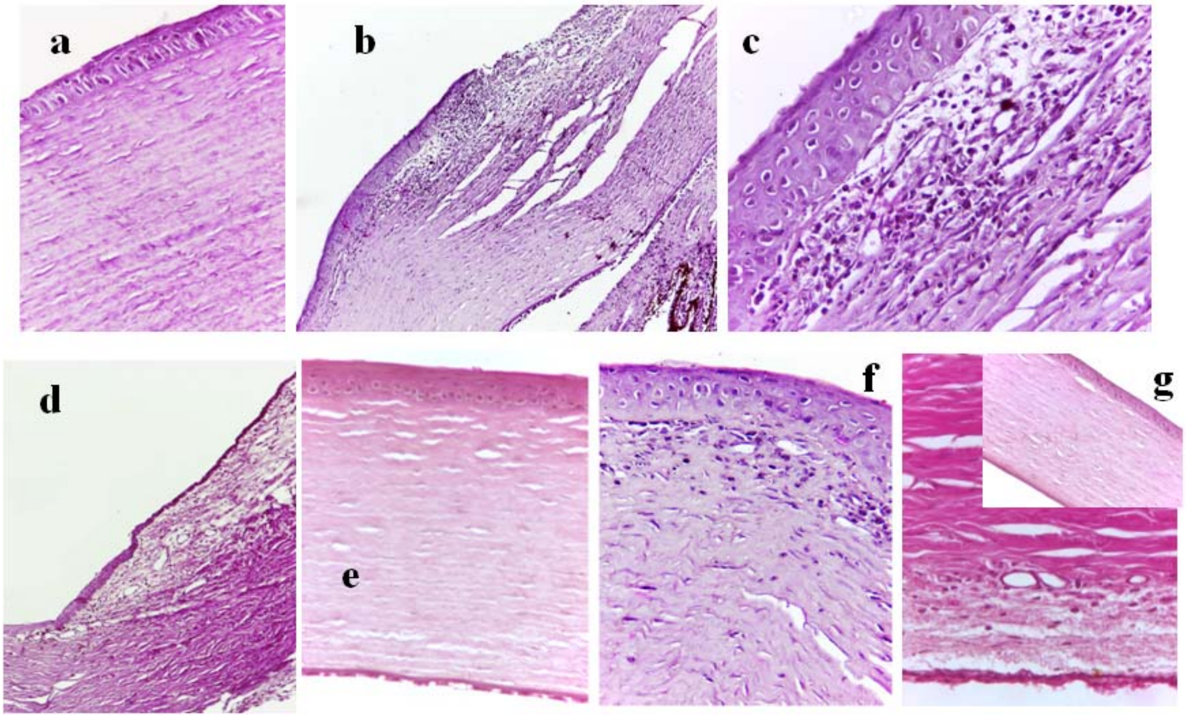

Supplementary Figure 2: Representative histopathological examples of corneas demonstrating cornea ulceration (a), necrosis (b), leukocyte infiltration density (c) in MRSA-inoculated animals and from (d) to (g) animals after treatment with chloramphenicol (d), aqueous extract of *C. procera* latex (e), aqueous extracts of *F. sycomorus* leaves (f) , and alcoholic extracts of *F. sycomorus* leaves (g).

EXCLI Journal 2015;14:747-757 – ISSN 1611-2156
Received: April 05, 2015, accepted: May 20, 2015, published: June 16, 2015

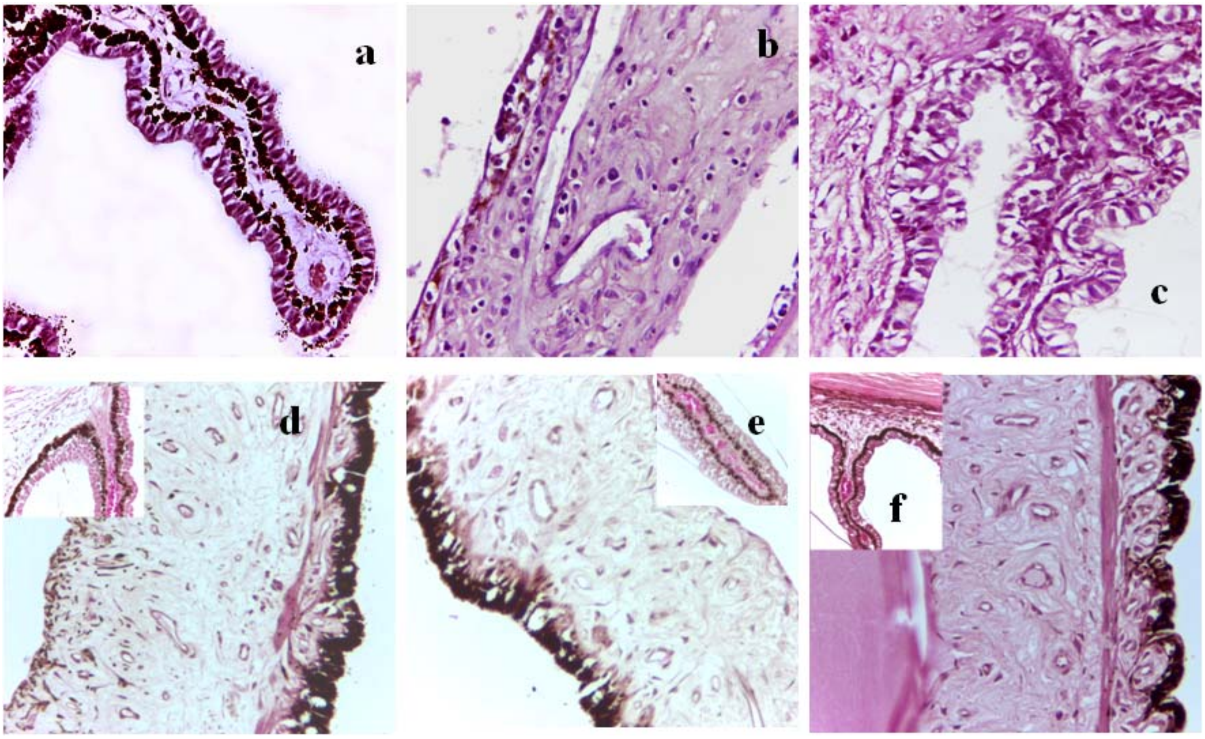

Supplementary Figure 3: Representative histopathological examples of iris, ciliary body and processes demonstrating edema (a), depigmentation and leukocytic infiltration (b) in MRSA-inoculated animals and from (c) to (f) animals after treatment with chloramphenicol (c), aqueous extract of *C. procera* latex (d), aqueous extracts of *F. sycomorus* leaves (e), and alcoholic extracts of *F. sycomorus* leaves (f).

EXCLI Journal 2015;14:747-757 – ISSN 1611-2156
Received: April 05, 2015, accepted: May 20, 2015, published: June 16, 2015

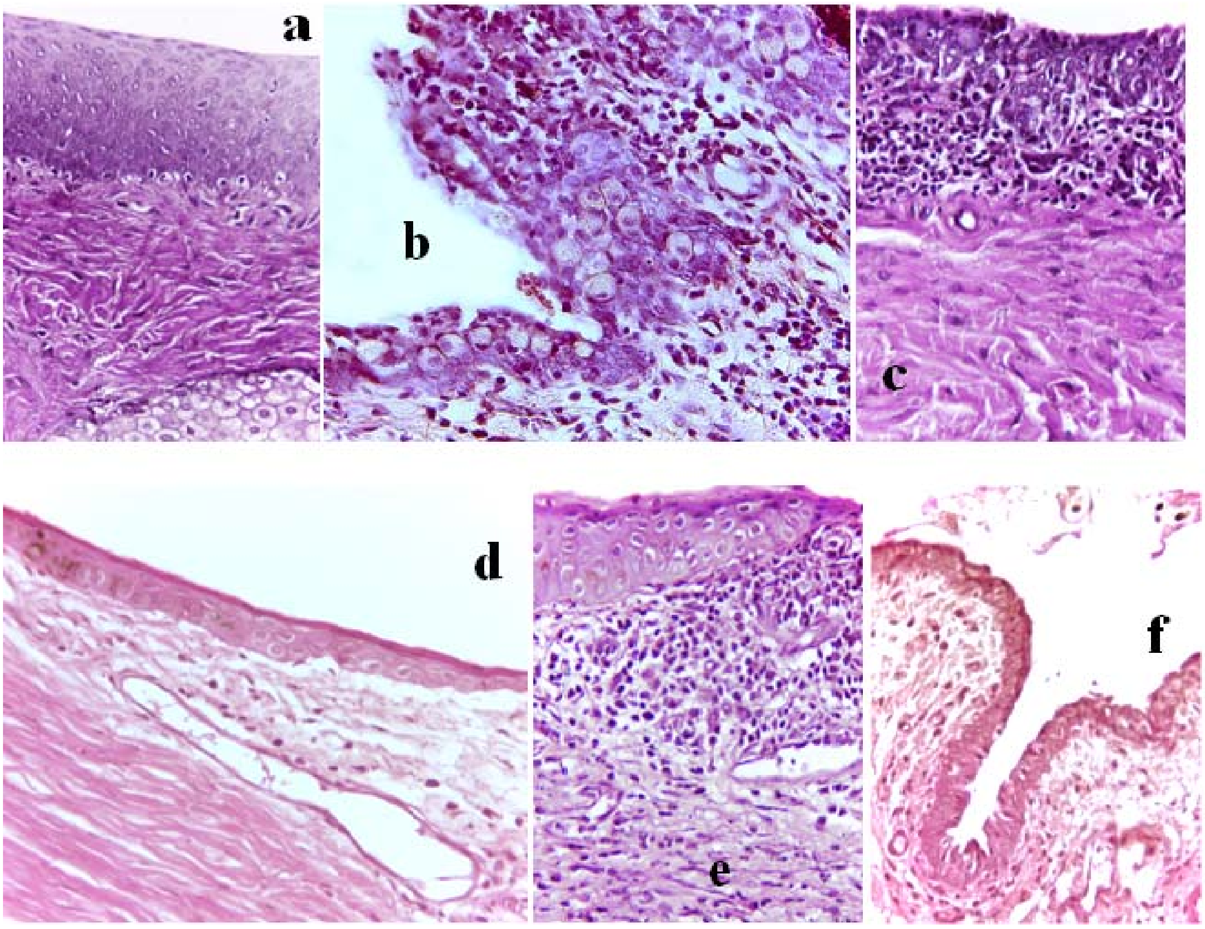

Supplementary Figure 4: Representative examples for histopathology of conjunctiva demonstrating: (a) normal tissue appearance of uninfected control, (b) mucosal necrosis and leukocytic infiltration in MRSA- inoculated animals, (c) after treatment with chloramphenicol, (d) after aqueous extract of *C. procera* latex, (e) and (f) after aqueous and alcoholic extracts of *F. sycomorus* leaves.
